# Supplementary material for: Evolving parsec-scale radio structure in the most distant blazar known
Source: Nat Commun. 2020 Jan 9;11:143. doi: 10.1038/s41467-019-14093-2 (PMC6952353; doi:10.1038/s41467-019-14093-2)
Supplement: Supplementary file 1 — Supplementary Information [file 41467_2019_14093_MOESM1_ESM.pdf]

Supplementary Information

Evolving parsec-scale radio structure in the most distant blazar known

Tao An[1*], Prashanth Mohan[1], Yingkang Zhang[1,2], Sándor Frey[3], Jun Yang[4], Krisztina É. Gabányi[5,3,6], Leonid I. Gurvits[7,8], Zsolt Paragi[7], Krisztina Perger[6,3], Zhenya Zheng[1]

1. Shanghai Astronomical Observatory, Key Laboratory of Radio Astronomy, 80 Nandan Road, 200030 Shanghai, China
2. University of Chinese Academy of Sciences, 19A Yuquan Road, Shijingshan District, 100049 Beijing, China
3. Konkoly Observatory, CSFK, Konkoly Thege Miklós út 15-17, H-1121 Budapest, Hungary
4. Department of Space, Earth and Environment, Chalmers University of Technology, Onsala Space Observatory, SE-439 92 Onsala, Sweden
5. MTA-ELTE Extragalactic Astrophysics Research Group, Pázmány Péter sétány 1/A, H-1117 Budapest, Hungary
6. Department of Astronomy, Eötvös Loránd University, Pázmány Péter sétány 1/A, H-1117 Budapest, Hungary
7. Joint Institute for VLBI ERIC (JIVE), Postbus 2, NL-7990 AA Dwingeloo, the Netherlands
8. Department of Astrodynamics and Space Missions, Delft University of Technology, Kluyverweg 1, 2629 HS Delft, the Netherlands

* Correspondence to Tao An, antao@shao.ac.cn

Supplementary Figures

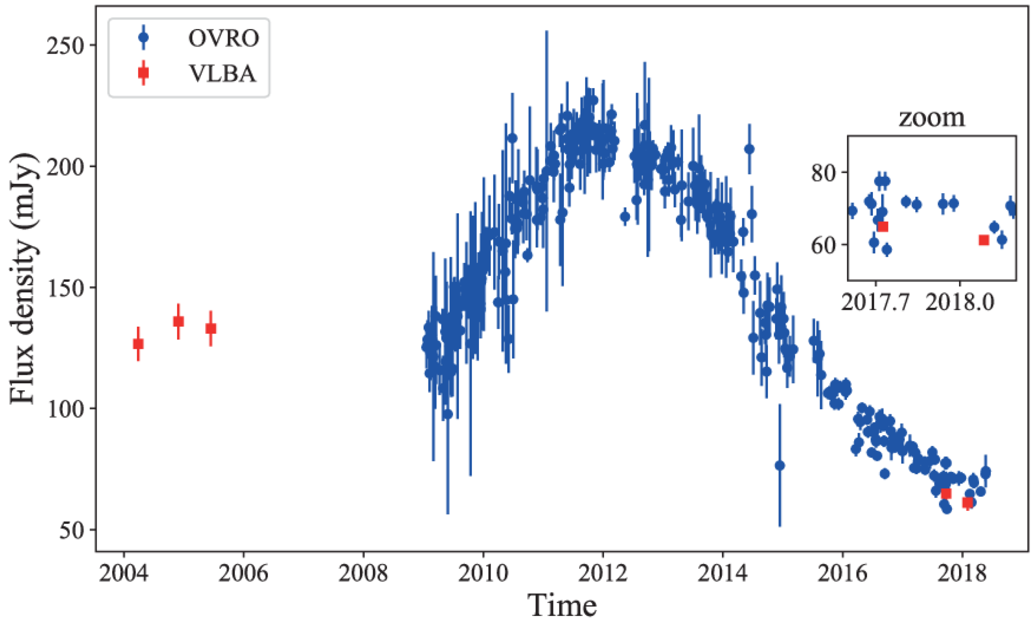

Supplementary Figure 1 The 15 GHz light curve of J0906+6930. The compiled and newly measured VLBA flux densities (shown as red points) are overlaid on the 15 GHz single dish flux densities (shown as blue points) from the Owens Valley Radio Observatory (OVRO). A prominent flare with a peak flux density of ~220 mJy occurs close to the end of 2011. The source flux density gradually decreases afterwards. The inset shows the latest epoch VLBI data points overlaid on the single-dish light curve, showing good consistency. The source flux density in 2017-2018 is at a minimum. Three earlier epoch VLBA data points are also plotted.

The error bars on the OVRO data points are based on the reported flux densities, and those on the VLBA points are based on the 1σ errors on the total flux densities (core and jet, see Table 1 of the main article).

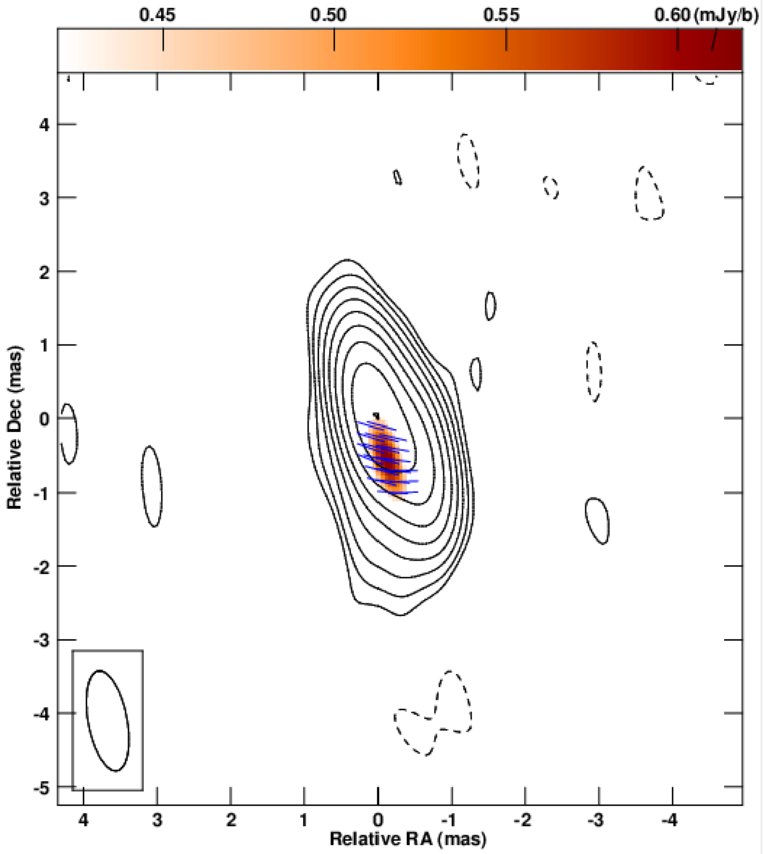

Supplementary Figure 2 The polarisation image of J0906+6930 (January 31, 2018). The color scale represents the linear polarisation, the unit of the colorbar is in mJy beam^{-1}. The Stokes I intensity image is shown as contours. The rms level in the image is 0.057 mJy beam^{-1}. The short blue-colored lines overlaid on the color gradients (dark orange shaded area) represent the uncalibrated electric vector position angle (EVPA) orientations. The length of the blue line denotes the polarisation intensity, 1 mas = 1.25 mJy beam^{-1}. The ellipse in the bottom left corner is the restoring beam, which is 1.38 mas × 0.53 mas, at a major axis position angle of 10.8°.

Supplementary Tables

NO.	Project code	ν (GHz)	Date	Time [a] (min)	Participating antenna [b]	Bandwidth [c] (MHz)	ref.
1	BR093	15.4	2004 Feb 27	75	VLBA all 10	16×2 (LCP+RCP)	1,2
2	BG154x	15.4	2004 Nov 22	120	VLBA all 10	16×2 (LCP+RCP)	3
3	BG154B	14.4	2005 Mar 22	40	VLBA 8 (no PT, SC)	64×1 (LCP+RCP)	2
4	BG154E	15.4	2005 May 15	120	VLBA 9 (no Br)	16×2 (LCP+RCP)	2
5	BZ068	15.2	2017 Sep 11	400	VLBA 9 (no SC)	128×2 (LCP+RCP)	3
6	BZ071	15.2	2018 Jan 31	80	VLBA 9 (no SC)	256×2 (LCP+RCP)	3

Supplementary Table 1. Observation setup and logs. a: The total observation time spent on the target source, in minutes. b: General VLBA observations contain the following ten telescopes: Brewster (BR), Fort Davis (FD), Hancock (HN), Kitt Peak (KP), Los Alamos (LA), Mauna Kea (MK), North Liberty (NL), Owens Valley (OV), Pie Town (PT), and Saint Croix (SC). One or two telescopes did not participate in some observations because of technical problem or maintenance. c: Total bandwidth of the recording backends, in MHz. LCP —left-handed circular polarisation; RCP — right-handed circular polarisation.

Figure Label	Project Code	Restoring Beam (θ_{maj}, θ_{min}, PA) (mas, mas, °)	S_{peak} (mJy beam^{-1})	rms (mJy beam^{-1})
1a	BR093	1.46×0.48, −0.79	121.4	0.180
1b	BG154x	1.36×0.45, 0.90	119.0	0.190
1c*	BG154B&E	0.73×0.55, −15.5	114.3	0.160
1d	BZ068	0.87×0.60, −14.2	40.0	0.034
1e	BZ071	1.38×0.53, 10.8	41.6	0.057

Supplementary Table 2. Image parameters. *: owing to their closeness in time, the data of BG154B and BG154E were combined to make a single image.

Epoch	$\sigma_{R, sta}$	$\sigma_{R, sys}$	σ_R
2004 02 27	0.018	0.055	0.058
2004 11 22	0.012	0.063	0.064
2005 comb	0.006	0.052	0.052
2017 09 11	0.001	0.049	0.049
2018 01 31	0.002	0.071	0.071

Supplementary Table 3. Positional errors of component J1.

Epoch	$\sigma_{R, sta}$	$\sigma_{R, sys}$	σ_R
2005 comb	0.033	0.055	0.061
2017 09 11	0.019	0.063	0.053
2018 01 31	0.028	0.052	0.076

Supplementary Table 4. Positional errors of component J2.

Comp.	μ(RA)	μ (Dec)	μ (R)
J1	–0.005±0.003	–0.004±0.003	–0.006±0.004
J2	0.010±0.005	0.019±0.005	0.019±0.006

Supplementary Table 5: Proper motion results (in unit of mas/yr). Note - the proper motion μ(R) is the square root of the sum of squares of μ(RA) and μ(Dec) . The significance of μ(Dec, J2) is larger than 3σ , roughly along the C-J2 direction, indicating the jet expansion.

Supplementary Note

We use new 15-GHz data observed with the VLBA in 2017 and 2018, archival VLBA data obtained in 2004–2005 (see details in Supplementary Table 1) and the flux densities reported by the 40 m telescope at the Owens Valley Radio Observatory (OVRO) to explore the evolution of the source morphology and infer its physical characteristics. The OVRO 15 GHz data enabled the verification of the amplitude calibration of the 15-GHz VLBA data. In Supplementary Figure 1 we plot the light curve together with our VLBA measurements in the 2017 and 2018 epochs. The new VLBA measurements are consistent with the single-dish flux densities (the inset of Supplementary Figure 1). This indicates that the integrated radio emission is dominated by the pc-scale compact core–jet. The dimming of the core and the brightening of J1 in 2017 and 2018 (in comparison to 2004 and 2005) possibly originates from the propagating shock (post the 2011 flare) interacting with the interstellar medium.

Although, as stated in the main text, our project was not designed to reconstruct the electric vector position angle (EVPA) of the yet to be detected polarised emission, once the detection was achieved, we reconstructed uncalibrated EVPA distribution following a standard procedure. EVPAs are calculated by using Stokes U and Q values, $\mathrm{EVPA} = \frac{1}{2}\tan^{-1}\frac{\mathrm{U}}{\mathrm{Q}}$. Supplementary Figure 2 represents the polarised emission image of J0906+6930 with uncalibrated distribution of EVPA. While specific orientation of the EVPA cannot be treated physically due to the absence of its calibration, the orderly distributed electric vectors are consistent with the shock-compressed magnetic field model.

The fitted Gaussian models are presented in Table 1. The positional errors of J1 and J2, as well as the derived proper motions are tabulated in Supplementary Tables 3–5.

References

1. Romani, R. W., Sowards-Emmerd, D., Greenhill, L. & Michelson, P. Q0906+6930: the highest redshift blazar. *Astrophys. J.* **610**, L9-L11 (2004).
2. Zhang, Y. et al. J0906+6930: a radio-loud quasar in the early Universe. *Mon. Not. R. Astron. Soc.* **468**, 69-76 (2017).
3. the present paper.
